# The value of protein induced by vitamin K absence or antagonist-II (PIVKA) in predicting the efficacy of transhepatic arterial chemoembolisation (TACE): A retrospective study

**DOI:** 10.5937/jomb0-59224

**Published:** 2026-01-28

**Authors:** Qing Shi, Jingui Xu, Zhuang Zhang, Xinxin Ruan

**Affiliations:** 1 Department of Hepatology, The First People's Hospital of Jiujiang City, China; 2 Department of Hepatobiliary Surgery, Peking University Shenzhen Hospital, China

**Keywords:** transhepatic arterial chemoembolisation (TACE), protein induced by vitamin K absence or antagonist-II (PIVKA), primary liver cancer, survival prognosis, retrospective study, transhepatička arterijska hemoembolizacija (TACE), protein izazvan nedostatkom vitamina K ili njegovim antagonistom-II (PIVKA), primarni rak jetre, prognoza preživljavanja, retrospektivna studija

## Abstract

**Background:**

To investigate the value of AFP combined with degamma-carboxylprothrombin (DCP), vitamin K absence or antagonist-II (PIVKA) in predicting the efficacy of transhepatic arterial chemoembolisation (TACE).

**Methods:**

The clinical data of 69 patients with hepatocellular carcinoma (HCC) who received TACE at our hospital between March 2020 and December 2024 were retrospectively analysed. Changes in the serum AFP and DCP levels of patients before TACE and after two consecutive TACE operations were analysed. The therapeutic effect of TACE was evaluated using the MRECIST criteria. The changes in AFP and DCP levels were compared with the imaging data from the same period (mRECIST criteria). The measurement data were tested for normality, and comparisons between two groups that conformed to a normal distribution were performed using two-way independent sample t-tests. The Mann-Whitney U test was used to compare normally distributed data between the two groups. The c2 test was used to compare the counting data between the two groups, and the Mann-Whitney U test was used to compare the rank-counting data between the two groups. Spearman correlation analysis was used to explore the correlation between mRECIST grade and AFP and DCP levels. The value of each index in the diagnosis of patients in the remission group was analysed by subject working characteristic curve analysis.

**Results:**

According to the mRECIST criteria, 38 patients were in the remission group, and 31 were in the nonremission group. After treatment, the AFP and DCP levels in the remission group were significantly lower than those in the non-remission group (Z=-3.366 and -4.065, P&lt;0.05). There were statistically significant differences in AAFP ADCP AAFP%, and ADCP% between the remission group and the nonremission group (Z=-4.837, -5.597, -4.210, and -5.851, respectively; P&lt;0.001). The mRECIST stage was negatively correlated with AAFP and ADCP (RS = -0.552 and -0.593, P&lt;0.001). The area under the working characteristic curve of AAFP% was 0.796, that of ADCP% was 0.912, that of AAFP% + ADCP% combined was 0.921, and that of AAFP% + ADCP% had the most significant diagnostic value.

**Conclusions:**

A combined analysis of serum AFP and DCP levels before and after TACE can be used to evaluate the therapeutic effect of TACE in patients with hepatocellular carcinoma.

## Abstract (sr)

**Uvod:**

Cilj je bio da se ispita značaj alfa-fetoproteina (AFP) u kombinaciji sa degama-karboksil-protrombinom (DCP), poznatim i kao protein izazvan nedostatkom vitamina K ili njegovim antagonistom-II (PIVKA-II), u predviđanju efikasnosti transhepatičke arterijske hemoembolizacije (TACE).

**Metode:**

Retrospektivno su analizirani klinički podaci 69 pacijenata sa hepatocelularnim karcinomom (HCC) koji su u periodu od marta 2020. do decembra 2024. godine lečeni TACE procedurom u našoj ustanovi. Analizirane su promene nivoa serumskih AFP i DCP vrednosti pre TACE-a i nakon dve uzastopne TACE procedure. Terapeutski efekat TACE-a je procenjivan korišćenjem mRECIST kriterijuma. Promene u nivoima AFP i DCP su upoređene sa snimcima iz istog perioda (mRECIST). Podaci su testirani na normalnost raspodele; poređenja između dve grupe sa normalnom raspodelom su rađena t-testom za nezavisne uzorke. Za poređenje podataka korišćeni su Mann-Whitney U test, C2 test i Spirmanova korelaciona analiza za ispitivanje povezanosti mRECIST stadijuma sa vrednostima AFP i DCP. Dijagnostička vrednost svakog parametra u grupi sa remisi-jom je analizirana ROC (receiver operating characteristic) analizom.

**Rezultati:**

Prema mRECIST kriterijumima, 38 pacijenata bilo je u grupi sa remisijom, dok je 31 pacijent bio u grupi bez remisije. Nakon tretmana, nivoi AFP i DCP u grupi sa remisijom su bili značajno niži u poređenju sa grupom bez remisije (Z=-3,366 i -4,065, P&lt;0,05). Postojale su statistički značajne razlike u vrednostima AAFP ADCP AAFP% i ADCP% između dve grupe (Z=-4,837, -5,597, -4,210 i -5,851, P&lt;0,001). Stadijum prema mRECIST kriterijumima je bio u negativnoj korelaciji sa AAFP i ADCP (RS = -0,552 i -0,593, P&lt;0,001). Povrsina ispod ROC krive za AAFP% je iznosila 0,796, za ADCP% 0,912, dok je kombinovana vrednost AAFP% + ADCP% bila 0,921, sto ukazuje na najvecu dijagnostičku vrednost.

**Zaključak:**

Kombinovana analiza serumskih nivoa AFP i DCP pre i posle TACE procedure se može koristiti za procenu terapijskog efekta TACE kod pacijenata sa hepatocelularnim karcinomom.

## Introduction

Hepatocellular carcinoma (HCC) ranks third among malignancy-related deaths in China [1] [2] [3]. Transhepatic arterial chemoembolisation (TACE) is widely recognised as the primary treatment for unresectable patients with advanced HCC, and it has emerged as the preferred nonsurgical treatment [4]. Serum tumour markers are bioactive substances, including abnormal gene expression products, that reflect the presence of tumours [5]. The detection of tumour markers in serum plays a crucial role in tumour diagnosis, staging, and prognosis. Alpha-fetoprotein (AFP) is a specific protein associated with primary liver cancer, and its levels are highly valuable for monitoring tumour efficacy and evaluating patient prognosis [6] [7] [8]. Des-gamma-carboxy-prothrombin (DCP), an abnormal protein resulting from vitamin K deficiency or antagonism, plays a significant role in the screening, early diagnosis, and prognosis of hepatocellular carcinoma (HCC) [9]. The combination of TACE and AFP can improve the early diagnosis rate of hepatocellular carcinoma, but few studies have evaluated the postoperative efficacy of TACE.

The role of vitamin K deficiency, or protein induced by vitamin K antagonism (PIVKA), in the treatment of liver cancer has received considerable attention [10]. Hepatocellular carcinoma (HCC) is a common malignant tumour worldwide, and transhepatic arterial chemoembolisation (TACE) has become an important treatment option for HCC [11]. However, predicting the efficacy of TACE has long been a challenging clinical problem, making it highly important to identify reliable predictors. The occurrence and development of liver cancer closely correlate with the PIVKA level, a specific liver cancer marker [12]. Vitamin K deficiency, or antagonist II deficiency, significantly increases PIVKA production, making it a crucial tool for liver cancer diagnosis and prognosis assessment [13] [14] [15]. However, there is no clear consensus on the value of PIVKA in predicting the efficacy of TACE.

Therefore, this retrospective study aimed to investigate the predictive value of PIVKA in the treatment of liver cancer patients with TACE. By systematically integrating existing research results, we will try to resolve the controversies in current research and provide a more reliable basis for clinical practice. This study aimed to provide guidance for the individualised management of TACE for liver cancer patients, improve treatment efficacy and patient prognosis, and thus improve patient quality of life.

## Materials and methods

### Research subjects

The data of a total of 69 HCC patients who received TACE alone in the department from March 2020 to December 2024 were retrospectively collected.

The inclusion criteria for patients were as follows: (1) had HCC diagnosed according to China's »Norms for Diagnosis and Treatment of Primary Liver Cancer (2023 edition)«; (2) had a United States Eastern Oncology Consortium (ECOG) physical fitness score of 0-2; (3) had Child Pugh grade A or B; and (4) had initial treatment involving TACE, and the number of consecutive treatments was 2.

The exclusion criteria for patients were as follows: (1) had diffuse HCC; (2) had an Eastern Cooperative Oncology Group (ECOG) score >2; (3) had decompensated cirrhosis (jaundice, ascites, gastrointestinal bleeding, hepatic encephalopathy); (4) had severe heart, brain, lung or kidney disease; (5) had taken vitamin K antagonists (such as warfarin) for the past 3 months; (6) had incomplete clinical data; and (7) did not conform to the TACE indications in the diagnosis and treatment standards for primary liver cancer.

The Ethics Committee of our hospital approved this study, and all included patients provided signed informed consent.

### Traditional TACE treatment

The Seldinger technique punctured the femoral artery, and then the short sheath of the 5F artery was successfully implanted. The catheter was inserted along the sheath into the superior mesenteric artery for indirect portal vein angiography, and the hepatic catheter was inserted into the proper hepatic artery for hepatic angiography to determine the extent of the tumour and the presence or absence of arteriovenous fistula. Oxaliplatin for chemical perfusion (100 mg, 50 mg/ bottle, Sanofi-Aventis France), epirubicin for embolisation (0.25 g/bottle, Pfizer (Wuxi) Co., LTD.) and iodine (10 mL/bottle, Shanghai Xudong Haipu Pharmaceutical Co., LTD.). Angiography was performed again after embolisation to observe the deposition of iodide ions in the tumour. If the blood flow rate remains high, add an appropriate amount of gelatin sponge particles (350-550 pm, Hangzhou Ailikang Medical Technology Co., Ltd.) until the blood vessel becomes residual after embolisation and angiography.

### Drug-carrying microspheres

TACE punctures the femoral artery using the Seldinger technique after successful insertion of a short sheath in the 5 F artery. The catheter was advanced along the sheath and then inserted into the superior mesenteric artery. Indirect portal venography was performed, and the hepatic duct was accessed through the proper hepatic artery for hepatic arteriography to determine the extent of the tumour and whether an arteriovenous fistula was present. Oxaliplatin (100 mg, 50 mg/bottle, Jiangsu Hengrui Pharmaceutical Co., Ltd.) was used for chemical perfusion; epirubicin (0.25 g/bottle, Shenzhen Wan Le Pharmaceutical Co., Ltd.) microspheres with a diameter ranging from 100 to 300 pm (Jiangsu Hengrui Pharmaceutical Co., Ltd.) suspension solution was made by mixing 10 to 15 mL evenly. After embolisation, angiography was performed again until the main supplying artery stopped flowing.

### Postoperative management

The patients received symptomatic treatment, including antiemetics, liver and stomach protection, and fluid supplementation. The AFP DCF, and imaging findings were reviewed 4-6 weeks after surgery.

### Data collection

Clinical data included AFP and DCP levels in HCC patients one week before TACE and 4 to 6 weeks after two consecutive TAces. Imaging data included findings one week before TACE treatment and four to six weeks after two successive surgeries.

### Short-term efficacy evaluation and grouping criteria

After TACE, 2 radiologists examined enhanced upper abdominal CT, MR, plain chest CT, or PET-CT images to evaluate the degree of tumour remission after TACE treatment according to mRECIST staging.

The therapeutic effect was categorised into four levels: complete response (CR), partial response (PR), stable disease (SD), and progressive disease (PD). CR and PR were classified as the remission group, and SD and PD were classified as the nonremission group.

### Statistical analysis

SPSS 23.0 statistical software was used for data analysis. The measurement data were tested for normality, and those conforming to a normal distribution were represented by the mean ± standard deviation (x̄±s). Two-way independent sample t tests were used for comparisons between two groups. Data that did not conform to the normal distribution were represented by the median and quartile distance [M(P25~P75)], and the Mann-Whitney U test was used for comparisons between the two groups. The χ^2^ test was used to compare the counting data between the two groups, while the Mann-Whitney U test was used to compare the rank-counting data between the two groups. Spearman correlation analysis was used to explore the correlation between the mRECIST classification and the AFP and DCP levels. Receiver operating characteristic (ROC) curves were used to analyse the diagnostic value of each index for therapeutic efficacy in the remission group, and P < 0.05 was considered statistically significant.

## Results

### Comparison of baseline data between the two groups

The study included 69 patients, with an average age of 60 years (range, 31-88 years), comprising 59 males and 10 females. According to the classification criteria, there were 38 patients in the remission group and 31 patients in the nonremission group. There were no significant differences in age, sex, hepatitis B status, Child Pugh grade, ECOG score, portal vein cancer thrombus status, number of extrahepatic metastases, tumour stage, number of traditional TACE procedures, or number of drug-loaded microsphere TACE procedures between the two groups (P>0.05) (Table 1).

**Table 1:** Comparison of baseline data between two groups of patients.

Index	Relief group (n=38)	Unresolved group (n=31)	Statistical value	P value
Gender (Male/Female)	31/7	28/3	x^2^=1.053	0.305
Age (Years)	61.55±13.31	58.10±11.65	t=1.134	0.261
Hepatitis B [Cases (%)]	23 (60.5)	16 (51.6)	x^2^=0.552	0.458
Tumour staging [Cases (%)]			Z=-1.807	0.072
I b	5 (13.2)	0 (0)		
IIa	1 (2.6)	0 (0)		
IIb	22 (57.9)	19 (61.3)		
IIIa	10 (26.3)	12 (38.7)		
Child Pugh grading [Cases (%)]			x^2^=0.464	0.496
A-level	28 (73.7)	25 (80.6)		
B-level	10 (26.3)	6 (19.4)		
ECOG score [Cases (%)]			x^2^=0.585	0.444
1	30 (78.9)	22 (71.0)		
2	8 (21.1)	9 (29.0)		
Portal vein cancer thrombus<br>[Cases (%)]	10 (26.3)	12 (38.7	x^2^=1.208	0.272
Extrahepatic metastasis<br>[Cases (%)]	1 (2.6)	0 (0)	x^2^=0.828	0.363
Received traditional TACE<br>treatment [Cases (%)]			Z=-0.288	0.773
0	6 (15.8)	6 (19.4)		
1	14 (36.8)	11 (35,5)		
2	18 (47.4)	14 (45.2)		
Received TACE treatment with<br>drug-loaded microspheres<br>[Cases (%)]			Z=-0.288	0.773
0	18 (47.4)	14 (45.2)		
1	14 (36.8)	11 (35.5)		
2	6 (15.8)	6 (19.4)		

### Analysis of changes in AFP and DCP levels

There were no significant differences in AFP and DCP levels between the two groups prior to treatment (P>0.05). The levels of AFP and DCP in the remission group were significantly lower than those in the nonremission group (P<0.05). There were statistically significant differences in ΔAFP, ΔDCP, ΔAFP%, and ΔDCP% between the remission group and the nonremission group (P<0.05) (Table 2).

**Table 2:** Comparison of AFP and DCP levels between two groups of patients before and after treatment.

Index	Relief group (n=38)	Unresolved group (n=31)	Z value	P value
Before treatment				
AFP (μg/L)	32.93 (5.59~922.52)	92.80 (9.93~1101.00)	-0.881	0.379
DCP (mAU/mL)	842.04 (104.51 4548.58)	581.23 (31.76~3046.79)	-1.200	0.230
After treatment				
AFP (μg/mL)	5.71 (2.57~75.55)	355.40 (10.18~2675.90)	-3.366	0.001
DCP (mAU/mL)	43.56 (16.56~512.63)	2359.05 (138.78~4678.90)	-4.065	<0.00
Difference before and<br>after treatment				
ΔAFP (μg/L)	4.06 (-0.32~795.50)	-186.02 (-1606.11~-0.25)	-4.837	<0.00
ADCP (mAU/mL)	564.52 (53.30~3557.15)	-302.47 (-2656.51~-10.73)	-5.597	<0.00
Percentage before and<br>after treatment				
ΔAFP%	45.45 (-12.64~95.88)	-76.18 (-249.29~-2.52)	-4.210	0.00
ΔDCP%	75.92 (45.71~95.37)	-114.92 (-474.54~-18.33)	-5.851	<0.001

### Correlation analysis of mRECIST staging with AFP and DCP

**Figure 1:**
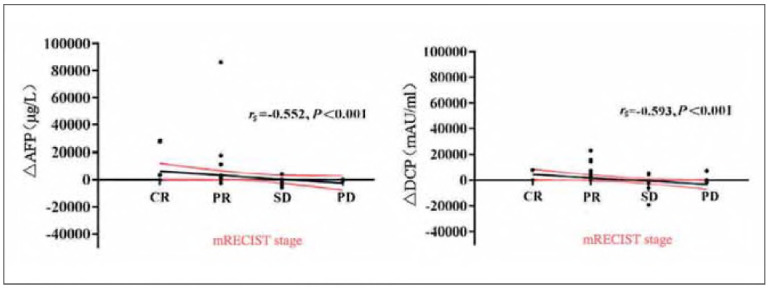
Scatter plot of mRECIST staging with ΔAFP and ΔDCP.

mRECIST stage was significantly negatively correlated with ΔAFP and ΔDCP (RS = -0.552 and -0.593, P<0.001) (Figure 1).

### Analysis of the ROC curves of the AFP and DCP groups

**Figure 2:**
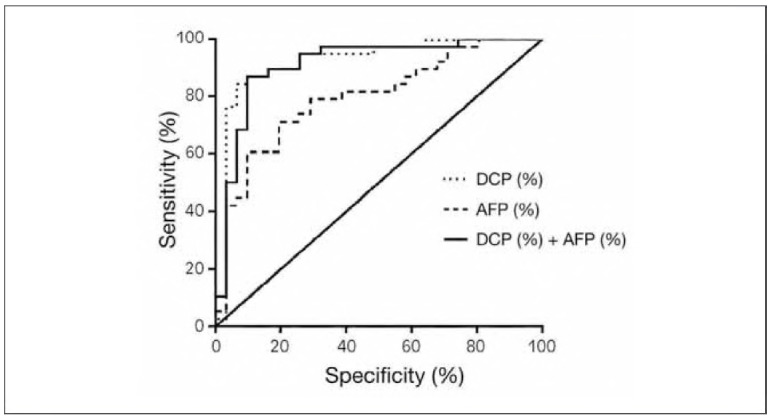
ROC curves of ΔAFP%, ΔDCP% and ΔAFP% + ΔDCP% for determining therapeutic efficacy in the remission group

The area under the ROC curve (AUC) of ΔAFP% was 0.796; that of ΔDCP% was 0.912, and that of ΔAFP% + ΔDCP% combined was 0. In total, 921, ΔAFP% + ΔDCP% had the highest diagnostic value (Table 3 and Figure 2).

**Table 3:** ROC curve analysis of treatment efficacy in the diagnostic remission group with ΔAFP%, ΔDCP%, and ΔAFP%+ΔDCP%.

Index	Area under<br>ROC curve	95% confidence<br>interval	Sensitivity (%)	Specificity (%)	P value
ΔAFP%	0.796	0.690~0.902	71.1	80.6	<0.001
ΔDCP%	0.912	0.836~0.987	86.8	90.3	<0.001
ΔAFP%+DDCP%	0.921	0.847~0.995	84.2	93.5	<0.001

## Discussion

Clinical practitioners widely use TACE, the preferred palliative therapy for HCC, as it can effectively improve patients' quality of life and prolong their survival [16] [17] [18]. Currently, the postoperative efficacy evaluation of TA-CE primarily relies on imaging methods (mRECIST standard), but this approach is expensive, and some patients exhibit poor compliance [19]. AFP is the most commonly used serological marker for the diagnosis of primary liver cancer, and it has a low detection cost and simple operation [20] [21] [22]. However, numerous transcription factors regulate AFP making it challenging to predict TACE efficacy in HCC patients [23] accurately. DCP is an abnormal prothrombin fluid that was first discovered by Liebman et al. The abnormal increase in DCP in the serum of HCC patients is closely related to the occurrence, development, invasion, and metastasis of HCC. It can be used as an indicator of the postoperative efficacy of TACE in HCC patients.

There are relatively few clinical studies [24] [25] [26] using AFP and DCP to evaluate the therapeutic effect of TACE. After TACE treatment, included 95 HCC patients, and the results demonstrated that both AFP and DCP could serve as indicators to assess the postoperative curative effect of TACE in HCC patients. When AFP is low, the predictive value of DCP is more advantageous than that of AFP It was found a significant decrease in the expression levels of OM, AFP, AFP-L3, and DCP in patients receiving effective TACE treatment, as well as an increase in the expression of these markers in patients experiencing relapse. In this study, the remission group had lower levels of AFP and DCP than did the nonremission group. The remission group also had higher levels of ΔAFP and ΔDCP than did the nonremission group. Both ΔAFP and ΔDCP were positive, indicating that they were classified as CR or PR according to the mRECIST criteria. Negative values of ΔAFP and ΔDCP correspond to SD or PD grades according to the mRECIST. The results showed that the tumour load decreased in the TACE remission group and increased in the nonremission group, as reflected by the decrease or increase in the serum AFP level. DCP also had a corresponding effect [27] [28] [29] [30]. Therefore, the tumour load can be predicted by monitoring AFP and DCP levels after TACE to predict the therapeutic effect.

The difference between AFP and DCP before and after TACE was examined in more detail [31]. The mRECIST stage was found to be significantly and negatively correlated with both AFP and DCP (rs<0, P<0.05) [32]. The decrease in AFP and DCP levels after TACE in HCC patients was associated with a better therapeutic effect, as determined by mRECIST staging, and a more favourable clinical therapeutic effect [33]. This study investigated the relationship between changes in the AFP and DCP levels before and after TACE treatment and the therapeutic effect. Still, the following limitations remain: (1) The small sample size prevented us from performing a subgroup analysis on traditional TACE and drug-loaded microsphere TACE, and the short-term efficacy of the drug-loaded microspheres was superior to that of conventional TACE, potentially biasing the overall results. (2) There was bias in both the human grouping and case selection processes. (3) The short follow-up time and short-term efficacy evaluation prevented further analysis of patient survival. Therefore, the relevant conclusions need to be confirmed by large sample sizes, multicenter studies, and prospective randomised trials.

## Conclusion

Our study revealed significant changes in serum AFP and degamma-carboxy prothrombin levels in patients with hepatocellular carcinoma after transcatheter arterial chemoembolisation. The level of AFP decreased after treatment, while the level of degamma-carboxyl-prothrombin increased. We found that AFP and degamma-carboxy-prothrombin have a certain predictive value for determining treatment efficacy in patients with HCC after TACE. Specifically, we found a positive correlation between decreased AFP levels after treatment and treatment effectiveness, while increased levels of degamma-carboxy-prothrombin also improved treatment effectiveness. These results suggest that AFP and degamma-carboxy-prothrombin serve as crucial markers for assessing the therapeutic impact of TACE.

## Dodatak

### Conflict of interest statement

All the authors declare that they have no conflict of interest in this work.

## References

[1] Deng M, Cai H, He B, Guan R, Lee C, Guo R (2023;Nov 1). Hepatic arterial infusion chemotherapy versus transarterial chemoembolization, potential conversion therapies for single huge hepatocellular carcinoma: A retrospective comparison study. Int J Surg.

[2] Hiraoka A, Kumada T, Kariyama K, Toyoda H, Yasuda S, Tsuji K, Hatanaka T, Kakizaki S, Naganuma A, Ishikawa T, Tada T, Takaguchi K, Itobayashi E, Shimada N, Shibata H (2022). Simple Scoring System for Predicting TACE Unsuitable among Intermediate-Stage Hepatocellular Carcinoma Patients in the Multiple Systemic Treatment Era. Oncology.

[3] Wang S, Su T, Chen B, Liu C, Liu C, Yang H, Tseng T, Chen P, Kao J (2022;Aug). Prothrombin induced by vitamin K absence or antagonist-II (PIVKA-II) predicts complete responses of transarterial chemoembolization for hepatocellular carcinoma. J Formos Med Assoc.

[4] Kumar A, Acharya S K, Singh S P, Arora A, Dhiman R K, Aggarwal R, Anand A C, Bhangui P, Chawla Y K, Datta G S, Dixit V K, Duseja A, Kalra N, Kar P, Kulkarni S S, Kumar R, Kumar M, Madhavan R, Mohan Prasad V G, Mukund A, Nagral A, Panda D, Paul S B, Rao P N, Rela M, Sahu M K, Saraswat V A, Shah S R, Sharma P, Taneja S, Wadhawan M, Shalimar, INASL Task-Force on Hepatocellular Carcinoma (2020;Jan-Feb). 2019 Update of Indian National Association for Study of the Liver Consensus on Prevention, Diagnosis, and Management of Hepatocellular Carcinoma in India: The Puri II Recommendations. J Clin Exp Hepatol.

[5] Wang L, Yang X, Wang J, Yu G (2023;Dec 1). Predictive value of PIVKA-II and AFP for the non-objective response of HBV-associated hepatocellular carcinoma after transarterial chemoembolization: A prospective study. Eur J Gastroenterol Hepatol.

[6] Wu L, Zheng Y, Liu J, Luo R, Wu D, Xu P, Wu D, Li X (2021;Apr 20). Comprehensive evaluation of the efficacy and safety of LPV/r drugs in the treatment of SARS and MERS to provide potential treatment options for COVID-19. Aging (Albany NY).

[7] Sawatsubashi T, Nakatsuka H, Nihei K, Takano T (2020;Feb). A Case of Metachronous Multiple Liver Metastases of AFP and PIVKA-Producing Gastric Cancer, Responding to Transcatheter Arterial Chemoembolization (Japanese). Gan To Kagaku Ryoho.

[8] Allaire M, Bruix J, Korenjak M, Manes S, Maravic Z, Reeves H, Salem R, Sangro B, Sherman M (2022;Sep 8). What to do about hepatocellular carcinoma: Recommendations for health authorities from the International Liver Cancer Association. JHEP Rep.

[9] Wu L, Zhong Y, Wu D, Xu P, Ruan X, Yan J, Liu J, Li X (2022;Sep 10). Immunomodulatory Factor TIM3 of Cytolytic Active Genes Affected the Survival and Prognosis of Lung Adenocarcinoma Patients by Multi-Omics Analysis. Biomedicines.

[10] Xi D, Xu M, Han M, Guan Q, Guo Q, Yan F, Yao J, Ning Q (2023). Novel Prognostic Nomogram to Predict Progression-Free Survival of Patients with Hepatocellular Carcinoma After Transarterial Chemoembolization. J Hepatocell Carcinoma.

[11] Hiraoka A, Ishimaru Y, Kawasaki H, Aibiki T, Okudaira T, Toshimori A, Kawamura T, Yamago H, Nakahara H, Suga Y, Azemoto N, Miyata H, Miyamoto Y, Ninomiya T, Hirooka M, Abe M, Matsuura B, Hiasa Y, Michitaka K (2015). Tumor Markers AFP, AFP-L3, and DCP in Hepatocellular Carcinoma Refractory to Transcatheter Arterial Chemoembolization. Oncology.

[12] Wu L, Liu Q, Ruan X, Luan X, Zhong Y, Liu J, Yan J, Li X (2023;Jun 29). Multiple Omics Analysis of the Role of RBM10 Gene Instability in Immune Regulation and Drug Sensitivity in Patients with Lung Adenocarcinoma (LUAD). Biomedicines.

[13] Sun H, Yang W, Zhou W, Zhou C, Liu S, Shi H, Tian W (2022;Dec 29). Prognostic value of des-γ-carboxyprothrombin in patients with AFP-negative HCC treated with TACE. Oncol Lett.

[14] Xu R, Ji X, Pei X, Yu Y (2023;Feb 15). Comparison of efficacy and safety between transarterial chemoembolisation (TACE) combined with lenvatinib versus TACE combined with sorafenib in the treatment of intermediate and advanced hepatocellular carcinoma. Am J Transl Res.

[15] Zhao S, Qiu L, Zhao H, Gu W, Yang X, Gu Z, Shi R, Ni C (2021;Jul). Prognostic nomogram for hepatocellular carcinoma patients after transarterial chemoembolization based on des-γ-carboxy prothrombin reactivity and modified Response Evaluation Criteria in Solid Tumors. J Cancer Res Ther.

[16] Kong C, Zhao Z, Chen W, Lv X, Shu G, Ye M, Song J, Ying X, Weng Q, Weng W, Fang S, Chen M, Tu J, Ji J (2021;Oct). Prediction of tumor response via a pretreatment MRI radiomics-based nomogram in HCC treated with TACE. Eur Radiol.

[17] Wang S, Zhang X, Chen Q, Jin Z, Lu J, Guo J (2023;Apr 20). A Novel Neutrophil-to-Lymphocyte Ratio and Sarcopenia Based TACE-Predict Model of Hepatocellular Carcinoma Patients. J Hepatocell Carcinoma.

[18] Wu L, Zheng Y, Ruan X, Wu D, Xu P, Liu J, Wu D, Li X (2022;Jan 1). Long-chain noncoding ribonucleic acids affect the survival and prognosis of patients with esophageal adenocarcinoma through the autophagy pathway: Construction of a prognostic model. Anticancer Drugs.

[19] Kaewdech A, Sripongpun P, Assawasuwannakit S, Wetwittayakhlang P, Jandee S, Chamroonkul N, Piratvisuth T (2023;May 2). FAIL-T (AFP, AST, tumor sIze, ALT, and Tumor number): A model to predict intermediate-stage HCC patients who are not good candidates for TACE. Front Med (Lausanne).

[20] Ji Q, Fu Y, Zhu X, Wang L, Ling C (2021;Jan-Feb). Effect of RFA and TACE combined with postoperative cytokine-induced killer cell immunotherapy in primary hepatocellular carcinoma. J BUON.

[21] Fan W, Zhu B, Yue S, Zheng X, Yuan G, Yu L, Huang W, Huang S, Wei W, Li F, Huang Z, Tang R, Fan H, Li Z, Qiao L, Huang F, Cheng Y, Zhang Y, Wu Y (2022;Apr). Identifying optimal candidates for post-TIPS patients with HCC undergoing TACE: A multicenter observational study. Eur Radiol.

[22] Kong L, Wei G, Lv T, Jiang L, Yang J, Zhao Y, Yang J (2021;Jan 12). Outcome of TACE treatment in HIV infected patients with hepatocellular carcinoma. Sci Rep.

[23] Liu K, Ding Y, Wang Y, Zhao Q, Yan L, Xie J, Liu Y, Xie Q, Cai W, Bao S, Wang H (2022;Jun). Combination of IL-34 and AFP improves the diagnostic value during the development of HBV related hepatocellular carcinoma. Clin Exp Med.

[24] Chuang Y, Cheng Y, Tsang L L, Ou H, Hsu H, Lim W, Huang P, Weng C, Yu C (2023;Jan 15). Efficacy and Safety of Combined Ethanol-Lipiodol Mixture and Drug-Eluting Bead TACE for Large HCC. J Hepatocell Carcinoma.

[25] Wu L, Zhong Y, Yu X, Wu D, Xu P, Lv L, Ruan X, Liu Q, Feng Y, Liu J, Li X (2022;Oct 1). Selective poly adenylation predicts the efficacy of immunotherapy in patients with lung adenocarcinoma by multiple omics research. Anticancer Drugs.

[26] Cao Z, Cheng Y, Wang J, Liu Y, Yang R, Jiang W, Li H, Zhang X (2021;Apr 1). HBP1-mediated transcriptional repression of AFP inhibits hepatoma progression. J Exp Clin Cancer Res.

[27] Yang H, Lu L, Guo W, Gong B, Wang X, Chen Y, Chen X (2024;Jan 25). A Longitudinal Study of AFP Trajectories and Clinical Outcomes in Intermediate-Stage Hepatocellular Carcinoma After Hepatectomy. J Hepatocell Carcinoma.

[28] Tang X, Zhang Y, Dong X, Jiang G, Hong D, Liu X (2023;Jul 18). The Synergy of Gene Targeting Drug Icaritin Soft Capsule with Immunomodulator and TACE Brings New Hope for Drug Combination in Patients with Advanced Liver Cancer: A Case Report and Literature Review. Cancer Manag Res.

[29] Ma K, Liu J, Wang Y, Zhong Y, Wu Z, Fan R, Guo S (2020;Dec). Relationship between plasma cell-free DNA (cfDNA) and prognosis of TACE for primary hepatocellular carcinoma. J Gastrointest Oncol.

[30] Wu L, Li H, Liu Y, Fan Z, Xu J, Li N, Qian X, Lin Z, Li X, Yan J (2024). Research progress of 3D-bioprinted functional pancreas and in vitro tumor models. International Journal of Bioprinting.

[31] Wang Y, Song Z, Guo X, Yin C (2021;Jan- Mar). Therapeutic efficacy of TACE 125I seed implantation and its combination with intra-tumor injection of cisplatin for the treatment of hepatocellular carcinoma. Indian J Cancer.

[32] Wu L, Li X, Qian X, Wang S, Liu J, Yan J (2024;Feb 12). Lipid Nanoparticle (LNP) Delivery Carrier-Assisted Targeted Controlled Release mRNA Vaccines in Tumor Immunity. Vaccines (Basel).

[33] Wu Z, Cui L, Qian J, Luo L, Tu S, Cheng F, Yuan L, Zhang W, Lin W, Tang H, Li X, Li H, Zhang Y, Zhu J, Li Y, Xiong Y, Hu Z, Peng P, He Y, Liu L, He K, Shen W (2023;Apr 7). Efficacy of adjuvant TACE on the prognosis of patients with HCC after hepatectomy: A multicenter propensity score matching from China. BMC Cancer.

[34] Sun Y, Xiong Y, Wang Q, Qiao W, Zhang H, Zhang Y (2023;Aug 28). Development and validation of a nomogram to predict the recurrence of hepatocellular carcinoma patients with dynamic changes in AFP undergoing locoregional treatments. Front Oncol.

[35] Wu L, Chen X, Zeng Q, Lai Z, Fan Z, Ruan X, Li X, Yan J (2024;Mar 28). NR5A2 gene affects the overall survival of LUAD patients by regulating the activity of CSCs through SNP pathway by OCLR algorithm and immune score. Heliyon.

[36] Yao W, Xue M, Lu M, Wang Y, Zhao Y, Wu Y, Fan W, Li J (2020;Dec 17). Diffuse Recurrence of Hepatocellular Carcinoma After Liver Resection: Transarterial Chemoembolization (TACE) Combined With Sorafenib Versus TACE Monotherapy. Front Oncol.

[37] Yang C, Luo Y, Yang H, Yao Z, Li X (2022;Jul 21). Effects of Early TACE Refractoriness on Survival in Patients with Hepatocellular Carcinoma: A Real-World Study. J Hepatocell Carcinoma.

[38] Lee M, Shin H P (2023;Dec 14). Efficacy of Transarterial Chemoembolization (TACE) for Early-Stage Hepatocellular Carcinoma. Medicina (Kaunas).

